# Nanotechnology in Transportation Vehicles: An Overview of Its Applications, Environmental, Health and Safety Concerns

**DOI:** 10.3390/ma12152493

**Published:** 2019-08-06

**Authors:** Muhammad Shafique, Xiaowei Luo

**Affiliations:** 1 Department of Architecture and Civil Engineering, City University of Hong Kong, Hong Kong; 2 Architecture and Civil Engineering Research Center, Shenzhen Research Institute of City University of Hong Kong, Shenzhen 518057, China

**Keywords:** nanotechnology, transportation vehicles, environmental concerns, human health, safety management

## Abstract

Nanotechnology has received increasing attention and is being applied in the transportation vehicle field. With their unique physical and chemical characteristics, nanomaterials can significantly enhance the safety and durability of transportation vehicles. This paper reviews the state-of-the-art of nanotechnology and how this technology can be applied in improving the comfort, safety, and speed of transportation vehicles. Moreover, this paper systematically examines the recent developments and applications of nanotechnology in the transportation vehicle industry, including nano-coatings, nano filters, carbon black for tires, nanoparticles for engine performance enchantment and fuel consumption reduction. Also, it introduces the main challenges for broader applications, such as environmental, health and safety concerns. Since several nanomaterials have shown tremendous performance and have been theoretically researched, they can be potential candidates for applications in future environmental friendly transportation vehicles. This paper will contribute to further sustainable research and greater potential applications of environmentally friendly nanomaterials in healthier transportation vehicles to improve the transportation industry around the globe.

## 1. Introduction

**Figure 1:**
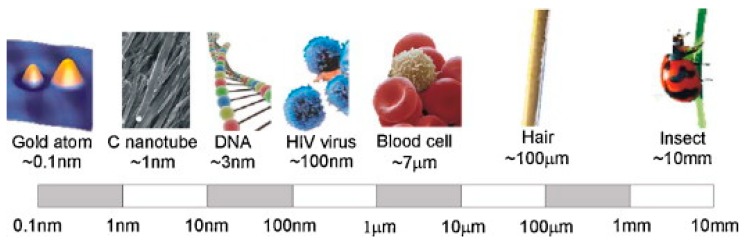
The representation of nanomaterials’ length measured in nanosize [1].

Nanotechnology is applied due to the unique material physical and chemical properties of its products, all of which make great contributions to the materials industries around the world. Nanotechnology can be defined as the manipulation of matter with a size ranging from 0.1 to 100 nanometers, as manifested in Figure 1 [1]. The physical and chemical properties unique to the nano-size can prompt miraculous efficiency enhancements in (photo) catalysis, optical sensitivity and mechanical strength, facilitating applications in energy storage and sensors, etc. Hence, the nanotechnology contributes to the development of tools, instruments, and structures by the controlled manipulation of shape and size measured in nanometers [2,3,4,5,6]. In fact, the particle size plays a very prominent role due to the fact the characteristics of the materials are inevitably affected at the nanometer scale. This is because at the nanoscale, the electrostatic forces and quantum effects overcome the forces of gravity and this leads to enhanced material properties. The nanotechnology revolution has had a ground-breaking impact in various fields such as chemistry, biology, and engineering [7,8,9,10]. The number of nanotechnology application is rising in different fields such as biomedicine, robotics, electronics, automobiles, and civil engineering industry (including transportation) because of their superior performance [3,11,12,13,14,15,16,17].

Recently, nanotechnology has been extensively applied in the transportation vehicles industry to bring novel functionalities and improve vehicle performance [18,19]. The application of nanomaterials in vehicles can provide better strength and durability performance over conventional materials. Nanotechnologies may offer new methods or tools for controlling or modifying the structures and properties of the materials to achieve better performance [20,21,22]. For the specific safety and durability of the vehicles (automobile, aerospace, and marines), nanomaterials have the potential to enhance vehicle safety due to their specific properties [23,24,25]. Moreover, to manufacture cost-effective and efficient vehicles, various nanomaterials, such as nanopowders and nanocoatings, are increasingly being used [18,25]. Within the transportation industry, coatings represent the largest portion of nanotechnology applications in which the optimal selection of nanomaterials can significantly enhance the sustainability of the coatings [18]. The primary objective of the application of nanotechnology is to create safe and sustainable transportation vehicles around the world.

In modern transportation, new smart high speed and efficient vehicles are unimaginable without the application of nanotechnologies such as lightweight nanomaterials, scratch resistant paints, and nanofluids. However, several studies [26,27,28,29,30] have manifested that the application of nanomaterials has some potential issues, including toxicity or exposure that can threaten living organisms as well as environmental health, and a precise framework and management system is needed to reduce these risks. The development of an effective and efficient regulatory scheme could be an effective strategy to classify nanomaterials based on their exposure and toxicity. Besides, a systematic management system could be adapted to hinder the application of toxic nanomaterials as well as to suggest the application of environmental friendly nanomaterials in real-world uses. This could reduce the environmental risks of nanotechnology in future developments around the world.

**Figure 2:**
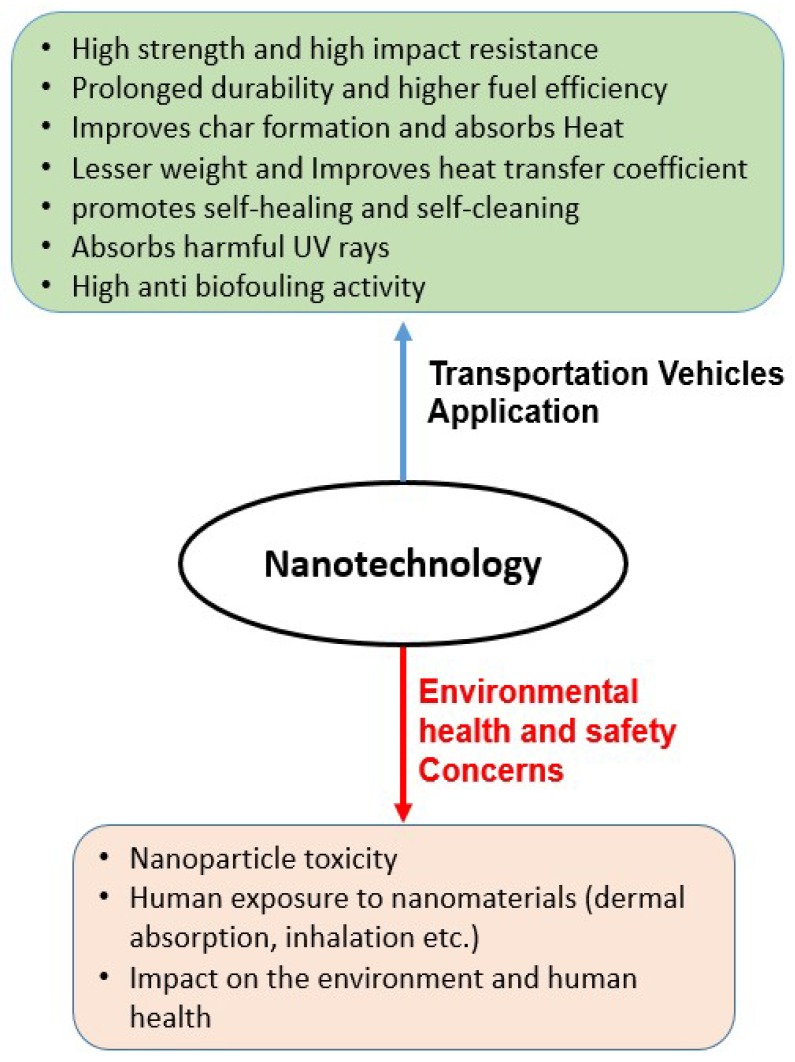
Potential applications of nanotechnology in transportation and the associated environmental health and safety concerns.

From a nanotechnology application in transportation vehicles viewpoint, various topics have been identified in the areas of nanotechnology application in vehicles [18,22,31]. Recent literature reviews [18,25] have summarized the potential benefits of nanotechnology in transportation vehicles, however, there are various environmental and safety concerns related to the application of nanotechnology [32,33,34]. From the literature research, it was found that there is a lack of comprehensive studies on nanotechnology applications in the transportation vehicles industry. In addition, there is a no comprehensive study which includes the overall application of nanotechnology with respect to environmental, health and safety concerns in vehicles (i.e., automobiles, marine vehicles, aerospace). This literature study aims to fill that gap, by comprehensively overviewing the applications of nanotechnology and the associated environmental concerns in transportation as shown in Figure 2. The main aim of this study is to provide a recent literature review on the application of nanotechnology in transportation vehicles which could provide useful information for sustainable and environmental friendly nanomaterials in the near future. This study stresses that the adaptation of environmental friendly nanomaterials could provide long term benefits in vehicles in the near future.

In this paper, we conduct an extensive literature review on the application of nanotechnology in vehicles and analyze its environmental, health and safety concerns. Through this analysis, we identify future opportunities for safer application of nanotechnology in vehicles that could yield a deeper understanding of how safer and more effective nano-based vehicles could be achieved. Section 2 describes the methodology used to select the appropriate literature. Section 3 identifies the applications of nanotechnology and their benefits in vehicles. Section 4 discusses the environmental and health concerns associated with the application of nanotechnology in transportation vehicles. Finally, Section 5 presents future perspectives for safer and more sustainable applications of nanotechnology in vehicles.

## 2. Literature Collection and Research Methodology

This literature study aims to provide a critical analysis of the state-of-the-art research into nanotechnology applications in vehicles concerning the environment, health and safety concerns. A thorough and extensive literature search can provide useful information regarding the basic knowledge about safer and sustainable nanotechnology applications in transportation vehicles and indicate future research directions. This methodology section consists of three steps, summarized as follows.

### 2.1. Selection of the Research Material

**Figure 3:**
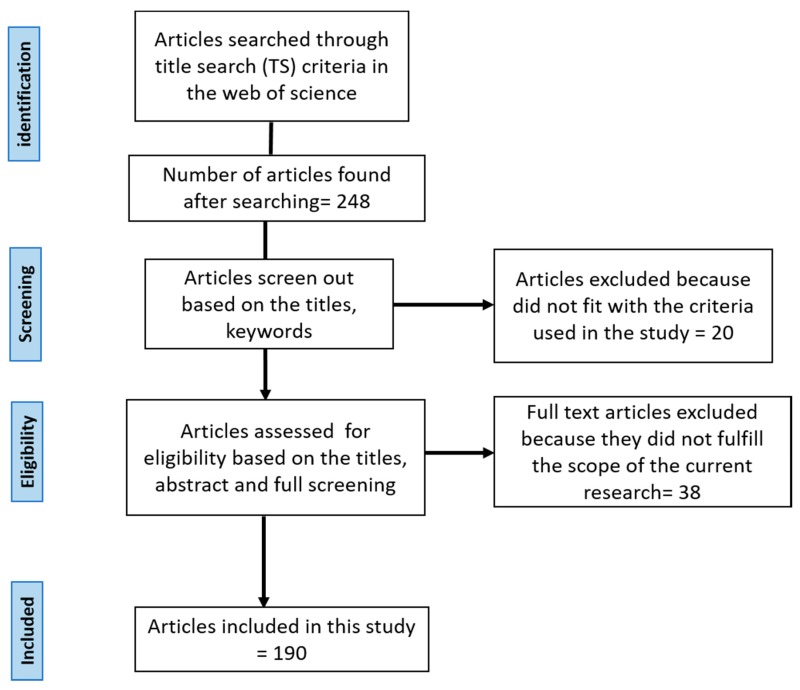
The flowchart diagram for screening articles for this study.

In the first step, all the articles published in reputable journals and proceedings which have a direct link with nanotechnology in transportation and the environment, health and safety (EHS) concerns of nanotechnology in transportation were selected and screened. First, papers were found through conducting searches in scientific databases (e.g., the Web of Science) using specific criteria. As this study focuses on the recent advancement of nanotechnology applications in transportation and the associated environmental concerns, the timespan of the search was set as from 2000 to 2018. For the initial Web of Science search, the criteria used was *TS* = ((nanotechnology AND (transportation OR nanotechnology in automobiles OR nanotechnology in aerospace OR nanotechnology in marine)) OR nanotechnology adverse effects OR nanotechnology consequences OR nanotechnology health concerns OR nanotechnology safety concerns OR nanotechnology challenges OR nanotechnology environmental concerns)) Indexes = SCI-EXPANDED, SSCI, A&HCI, CPCI-S, CPCI-SSH, BKCI-S, BKCI-SSH, ESCI Timespan = 2000–2018. Two hundred forty-eight articles were retrieved in the Web of Science. The initial selection of the articles to decide whether the articles are related to our scope or not was based on the titles, abstract and keywords as shown in Figure 3. Articles were selected if they were relevant to nanotechnology applications in transportation vehicles as well as associated with EHS concerns. 

### 2.2. Content Analysis Method

After the initial screen, the next step was to develop a content analysis method for the collection of relevant papers that dealt with our current topic. Content analysis is a research method which is widely used in the social science field [35], and it examines the information trends of a particular topic. After the initial step, we have analyzed all 248 articles based on their abstracts and keywords to only keep articles related to nanotechnology in transportation and associated EHS concerns. This step helped exclude around 20 articles which were not related to these topics, reducing the number of articles to 228. In addition, duplicate papers were excluded in this step. Moreover, we manually reviewed the full articles and papers which dealt with nanotechnology EHS in fields other than vehicles were excluded. Finally, 190 articles were retained as the subjects for this study. as shown in Figure 3. This study excluded articles related to nanotechnology in concrete, while it considered articles associated with the potential opportunities for cost-effective nanomaterials in transportation towards a safe, sustainable and smart city.

### 2.3. Validity of the Literature Research Process

This literature review presents the most relevant conference proceedings, expert reports and peer-reviewed journal articles in the field of nanotechnology and transportation vehicles. We followed a structured process to ensure a rigorous analysis of recent literature. We have included the content analysis method used to systematically select the most relevant articles for this review paper. 

## 3. Nanotechnology Applications in Transportation Vehicles

Nanotechnology has been applied in the transportation for multiple benefits, such as to enhance the strength and durability of automobiles over a longer period of time [18,25]. Nanotechnology could be applied to various body parts, including the chassis, tires, windows, engines, etc., to enhance their performance and durability [18]. However, there are certain health and environmental risks associated with the application of nanotechnology in transportation vehicles which demand high attention [36,37]. Therefore, this review study is divided into two parts. First it presents the nanotechnology applications in vehicles, and secondly, it identifies the nanotechnology-associated EHS concerns, so in future development, this study could serve as a guideline to adopt safer and sustainable nanomaterials in the vehicle industry. The details of nanotechnology applications in vehicles are described below.

**Figure 4:**
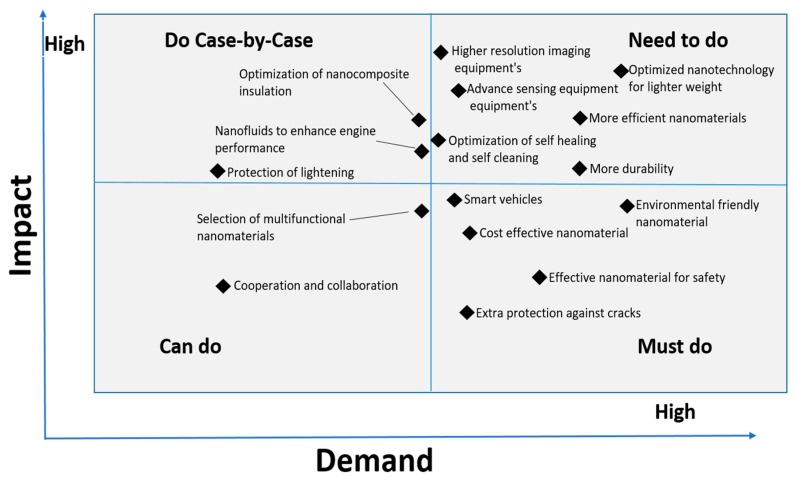
Impact of and demands for nanotechnology applications in the transportation vehicle industry.

The potential applications of nanotechnology in transportation vehicles are nearly endless. The design and production of nanomaterials, nanostructures, and nanodevices provide new ways for developing sustainable vehicles. Nanotechnology is used as a useful technology to provide protection for automobile bodies against corrosion and abrasion resistant. The impact of and need for nano-technological application in the transportation vehicle are manifested in Figure 4. For this purpose, the authors have searched for the most prominent factors which have a greater effect on the performance of transportation vehicles for a longer time. Then the impact and demand of those factors in the vehicles industry were identified in Figure 4. It is indicated that the factors such as the lighter weight nanotechnology and more efficient materials have high impact and high demand for the future of safer and more sustainable vehicles around the world. Figure 4 is segmented into four parts according to the demand and impact upside that can optimize the nanotechnology characteristics in transportation. It includes the segment “must do” which comprises of the use of more smart, cost-effective and environmentally friendly nanomaterials for the safe and sustainable application of nanotechnology in transportation. Similarly, the segment “need to do” was developed, which has a high impact and demand in the transportation industry. It includes optimization of nanotechnology for lighter weight, optimization of nanomaterials for self-cleaning and self-healing; and high sensing and high-resolution equipment. On the other hand, the other two segments, “do case-by-case and can do” could also have a high impact with moderate demand in the transportation industry for the enhancement of nanotechnology efficiency. The selection of multifunctional nanomaterials and optimization of nanocomposite insulation are also important aspects which could have high impact to make the transportation systems safer and more durable in the near future. Besides, there is a high demand for the selection of environmentally friendly nanomaterials in transportation because this will have high impact to create a more reliable and sustainable environment in the nano-industry around the world. The details of the applications of nanotechnology in transportation are explained below.

### 3.1. Nanotechnology Applications in the Automotive Industry

Nanotechnology can be incorporated in various automobile parts such as paint, batteries, fuel cells, tires, mirrors, and windows. The introduction of nanotechnologies enhances the performance of existing technologies for the automobile industry. The main advantages of applying nanotechnology in automobiles include providing lighter and stronger body parts (to enhance safety and fuel efficiency), improving fuel consumption efficiency, and therefore achieving a better performance over a longer period.

#### 3.1.1. Effective and Efficient Nano-Based Coatings for Automobiles

The coating with nanoparticles is an effective practice to enhance the protection and scratch resistance of automotive bodies [38,39]. Moreover, it also improves the appearance and provides durability over a longer period. Generally, the thickness of the outermost layer of the coating varies from 5–50 µm, and it is usually responsible for protecting the underlying layers from outside weather conditions and also improving the scratch resistance [21]. As vehicles are exposed to extreme weather conditions which can cause scratches and cracks on the body surfaces, under these circumstances, nano-based coatings proved as an effective strategy to protect the outer surfaces in such extreme weather conditions.

**Figure 5:**
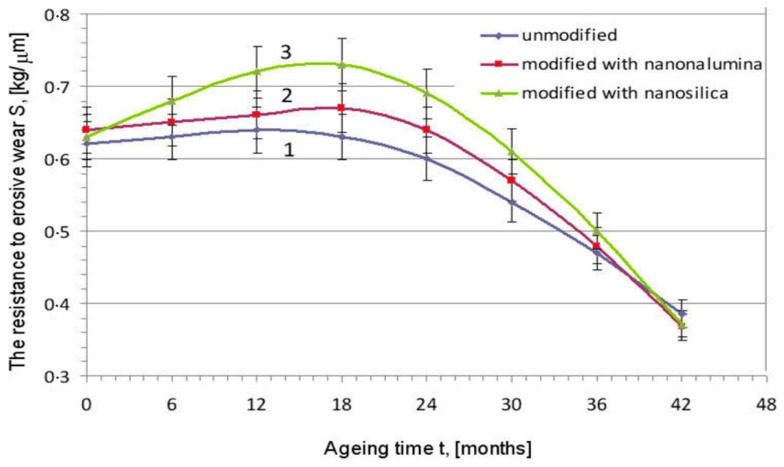
Representation of resistance to erosive wear of various coatings against aging time [46].

Several researchers [40,41,42,43] have identified that incorporation of nanoparticles in polymer coatings is responsible for upgrading the level of scratch and friction resistance to wear. This is because the presence of the nanoparticles in the coating layers improve the hardness, which protects them against cracking, wear and abrasion [43,44]. The study of [41] revealed that the additions of nano-SiO_2_ in the polymer coating could enhance the abrasion and scratch resistance, hardness and strength. Moreover, other nanoparticles such as SiC, ZrO_2_, ZnO, Al_2_O_3_, and TiO_2_ were also used for the enhancement of the coating properties [45,46,47]. Kotnarowska et al. [46] evaluated the performance of the unmodified epoxy-polyurethane coating and epoxy-polyurethane coatings modified with silica or alumina nanoparticles over three years. From the result analysis, it was found that the modified epoxy-polyurethane coatings indicated higher erosive wear resistance than the other two coatings as shown in Figure 5. Research also indicated that the nanoparticles inside the coating layer filled the pores and suppressed the development of cracks over the time interval [46].

**Figure 6:**
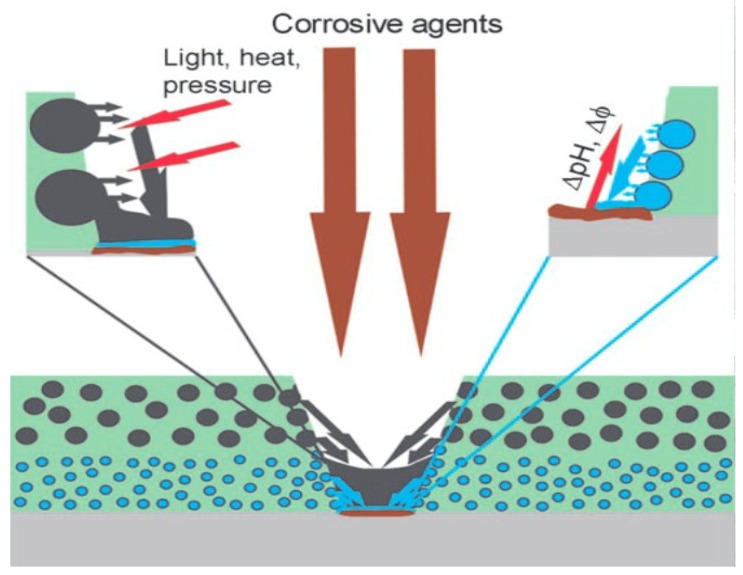
Nanomaterial self-repairing mechanism for surface coatings [50].

Furthermore, self-repairing coatings are adapted to increase the anti-corrosive properties of metals. In traditional coatings, the main purpose is to protect the surface against the corrosion. However, the coating performance degrades after a certain period. Under these circumstances, application corrosion inhibitors are an effective method to make an active coating when exposed to corrosive electrolytes. These corrosive agents are soluble in the corrosive electrolytes, which further protects the metal surface by a passivation process [38]. Cathodic inhibitors, anodic inhibitors, and mixed inhibitors are used for this purpose [48,49,50]. In the case of osmotic pressure, water or air transportation into the nanomaterial coating may destroy the passive coating layer. In this approach modern coatings with inhibitors could release the agent in the coating matrix [50]. As a result of this, the active agent leads the self-repairing activity of the coating as shown in Figure 6 [50].

Several studies [20,47,51,52] have indicated that the addition of nanoparticles in coatings tremendously improves the scratch and abrasion resistant over a longer period. However, for the optimal benefits, there is a need to select the appropriate proportion of nanoparticles in polymer coatings [47,53]. Ching and Syamimie [47] performed experiments to evaluate the optimal proportion of nano-silica in coatings for higher abrasion resistance. For these purposes, tests using 0 wt%, 3 wt% and 7 wt% of nano-SiO_2_ in the polyurethane coating were conducted. From the analysis of the results, it was found that a nano-SiO_2_ coating with 3 wt% content exhibits higher abrasion resistance. Therefore, the selection of the optimum proportions of nanoparticles in coatings is essential to enhance the strength and durability of the coatings over a longer period. In addition, to reduce the glass scratch problems of automobiles, nanomaterials can be embedded in polycarbonate polymeric glass by using polysiloxane or acrylate paints over automobile headlights. In the automotive clear coat, an organic matrix can be added using a sol-gel technique to enhance the scratch resistant [54]. This process can help adhesion resistance, and the inorganic coating phase could prevent mechanical damage to the automotive surface [54]. Similarly, [55] indicated that the addition of Al_2_O_3_ nanoparticles in the coating could help to enhance the abrasion resistance. This is because this glass coating makes the glass highly transparent due to the small size of the filler particles [55].

In comparison to traditional paints, nanomaterial-based paints show higher scratch resistance and great aesthetic value. The reason is that the addition of nanomaterials improves the coating properties and enhances the performance. However, in the future, there is still a need to find more cost-effective nanomaterial-based coatings for better scratch resistance performance over a longer period.

#### 3.1.2. Nanotechnology for Lightweight and Higher Strength Automobile Bodies

Incorporation of nanotechnology in the automotive industry helps to make automobiles safer, more durable and sustainable. The first utmost benefit of nanotechnology applications is that lighter and higher strength materials can be achieved [31,56,57]. As a result of the weight reduction of automobiles the fuel consumption could decrease tremendously. In addition, it helps to improve \ CO_2_ emission reductions in urban areas. Moreover, new advanced green lightweight materials for vehicles will only help vehicle reliability as well as fuel efficiency over a longer period [53].

The overall cost of the vehicle can be reduced by selecting appropriate nanomaterials in the automobile industry. This is because the cost is directly related to weight reduction. Moreover, the cost could be reduced more by selecting more cost-effective nanomaterials at the production stage [19]. Several studies [58,59] have indicated that carbon nanotubes (CNTs), clay nanocomposites with polyamide (PA), Mg, Al, Si, and TiO_2_ nanomaterials have lighter weight and have higher thermal properties which can enhance the overall strength and durability of automobiles over a longer period.

Furthermore, to enhance passenger safety in case of accidents, higher strength steel has been adopted for vehicles [38]. However, it is tough to recast high strength steel in the cold state because of a change in size and spring-back effects. Recasting at a higher temperature around 1000 °C helps to avoid such adverse circumstance [38]. To recast the steel at higher temperature nanotechnology coatings can be applied. For this purpose, recent multifunctional coatings are formed using aluminum particles combined with connected and bonded nano-sized vitreous and plastic-like materials. This process will provide higher strength and safety to vehicles during their operation in the real world. Lighter weight vehicles provide a faster and smoother ride and crash protection which helps safe and sustainable vehicle operation on the road.

#### 3.1.3. Safer and Secure Mirrors and Windows

**Figure 7:**
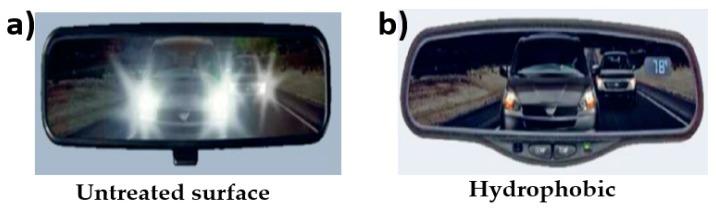
Representing the surface properties on glass plates in (**a**) conventional mirror (untreated surfaces) and (**b**) modern antiglare mirror (hydrophobic) [38].

Recently, the application of an ultra-thin reflective layer of aluminum oxide with thicknesses below 100 nm on the glass of vehicles is a very useful approach to enhance safety and security. During the day and night driving time, the discomfort (sunlight and the glare of the lights of oncoming vehicles) during driving could be significantly decreased by the application of ultra-thin reflective mirrors on vehicles [38,60]. An ultra-thin layer of aluminum oxide provides the mirror surface with dirt and water repellant features. These so called hydrophobic and oleophobic nanometer-thick layers are prepared by using the chemical vapor deposition (CVD) technique [60]. This technique is advantageous to enhance safety in the automobile industry. Figure 7a shows a conventional mirror while Figure 7b shows a modern hydrophobic layer-based mirror.

#### 3.1.4. Efficient and Durable Nano-based Tires

To ensure the safe operation of automobiles, there is a high need for the adoption of advanced nanomaterial-based tires. Typically, tire performance mainly depends on the cover composition, so the rubber composition of the tire cover significantly affects its overall long-term performance [38,61]. The proper rubber composition enhances the car safely on the road [38,60]. The addition of appropriate nanoparticles in rubber composites shows a positive impact on the safety and durability of tires [61]. Ref. [61] the study revealed that the addition of nano-Al_2_O_3_ in rubber composite enhances wear resistance over time. In addition, it also showed that nano-Al_2_O_3_ (2.5%) with carbon black (60 phr) tremendously improved the wear rate by up to 800%, hence enhancing the safety and durability of the tires in a real application. However, in the near future, there is a need to find effective nanomaterials which could enhance the performance of tires over longer periods.

#### 3.1.5. Nanotechnology for an Efficient Engine

**Figure 8:**
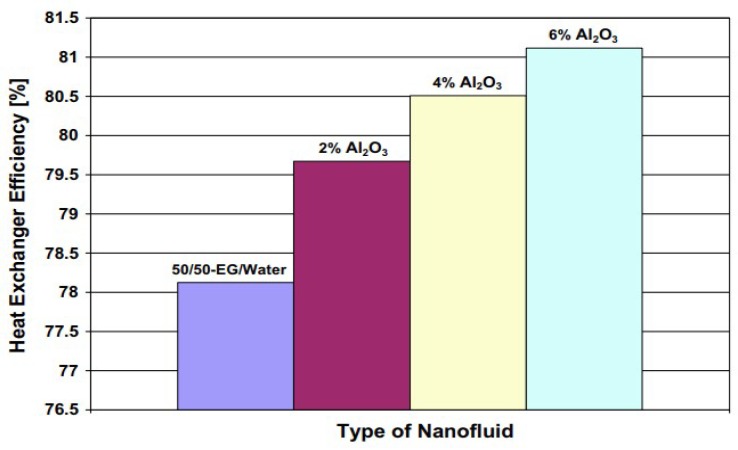
Illustration of the effect of various concentrations of Al_2_O_3_ nanofluid on the heat exchange efficiency of heat recovery of an engine [66].

Recently, for the environmental friendly technology the most highlighted issue in the transportation industry is the reduction of pollutants emissions from the engines [62,63]. For this purpose, a coating of aluminum nanomaterials could be used to reduce the friction of the cylinder walls [62,64]. Reference [65] conducted an experiment by adding the Al_2_O_3_ nanoparticles at various temperatures. Results showed that addition of nanomaterial enhanced the thermal conductivity by about 4.5% and 4.2% at temperatures of 50 °C and 30 °C respectively. Nonetheless, during the operation, the maximum amount of Al_2_O_3_ nanoparticles was about 1.5 vol% [65]. Another experimental study [66] revealed that with increased Al_2_O_3_ nanoparticle concentration, the heat exchange efficiency of the engine is increased, as shown in Figure 8. This also indicated that with the enhancement of Al_2_O_3_ nanoparticle concentration the cooling effect of the engine will be enhanced.

Furthermore, several experimental studies [67,68,69] have indicated that the addition of nanomaterials significantly improved the thermal conductivity of the vehicle engine. As a result of this, the fuel efficiency of the vehicle engine is enhanced significantly [70,71]. For example, the experimental study described in [72] revealed that the addition of 1.0 vol% of Al_2_O_3_–water nanofluid improved the coolant heat transfer coefficient, heat transfer rate and Nusselt number by about 14.7%, 14.8%, and 9.5%, respectively. In addition, another study indicated that the 0.4 vol% of SiO_2_–water maximized the heat transfer by around 9.3% as compared to the pure fluid [73]. Table 1 below compares the cooling performance of various nanofluids.

Nanofluid addition not helps to improve the thermal conductivity of the vehicle engine, but also helps to reduce pollutant emissions [75,76]. An experimental study [75] was performed by adding silver nanoparticles to pure diesel fuel. From the result evaluation, it was concluded that the inclusion of the nanomaterial greatly reduced the emission rates of NO_x_ and CO by up to 13 and 20.5%, respectively [75]. Similarly, the study of [76] indicated when aluminum nanoparticles are mixed with diesel fuel, it can reduce smoke concentrations significantly as compared to simple diesel fuel. Therefore, the additions of nanomaterial in the engine of the vehicle also contributes to reducing the emissions of hazardous gases.

#### 3.1.6. Nanotechnology Applications for a Safer Indoor Environment in Vehicles

For a safer and clean environment inside the automobile, careful attention is needed to reduce various bacteria and microbial diseases. This can be done by choosing environmentally friendly nano-agents such as gold, titanium oxide, silver, liposomes loaded with nanoparticles, titania nanotubes and copper [77,78,79,80]. These nanoparticles are very effective to provide a healthier environment inside the automobile. For example, gold and silver nanoparticle-based antimicrobial agents are biocidal. The microorganisms are usually destroyed through the interaction between the negatively charged cell membrane of the microorganism and the positively charged biocide.

Similarly, silver nanoparticles work as antibacterial agents due to their high degree of biocompatibility [38,80]. For the high-quality interior air quality of the automobile, novel filters covered with nanofibers are proved an effective strategy [60]. On the other hand, several studies have also indicated that nanomaterials could work as flame retardant agents to enhance occupant safety in the case of accidents [81,82,83]. For example, CNTs and silver nanomaterials can be used as a filler in automobile fabrics to reduce the chances of fire [84,85]. The incorporation of these nanomaterials into fabrics makes them less ignitable as compared to regular fabrics. Therefore, through careful consideration nanomaterials could be applied in the interior of automobiles.

### 3.2. Nanotechnology Applications in the Aerospace Industry

Apart from automobiles, applications of nanotechnology have been proved a sustainable approach for aerospace uses due to their higher tensile strength and lighter weight [86,87,88]. This will not only reduce the overall weight of the aircraft but also decrease the fuel consumption. Next generation aircraft require light weight, higher speed, and maneuverability [87]. CNTs are the optimal approach to fulfil these requirements, as they are multifunctional. Carbon nanotube applications include lower weight, higher tensile strength, removal of CO_2_, icing mitigation and electromagnetic shielding on aircraft, contributing to effective wing materials and lubricants [89,90]. Apart from strength, CNTs are electrically conductive materials which help enhance the conductivity of composite panels which permits current to move throughout the whole structure of the airplane [88]. This further protects the aircraft against electrical discharge accidents [88]. Aerospace applications require high perfection and security as a tiny defect/error in operation will risk the lives of the passengers [86]. Therefore, there is a need for materials which have high tensile strength, as well as higher resistance to corrosion and fire [86]. Another major concern which requires great attention is a selection of lightweight materials for aerospace [86,91]. The application of nanotechnology can provide effective and sustainable techniques for aerospace applications with higher tensile strength, less weight, less fuel consumption and also in advanced filters for air purification [91]. The application of suitable nanomaterials in various parts of the aircraft could enhance its overall performance over a longer period. Potentials applications of nanotechnology in the aerospace industry are explained below.

#### 3.2.1. Nanotechnology Applications for Higher Strength and Lighter Weight in Aerospace

**Figure 9:**
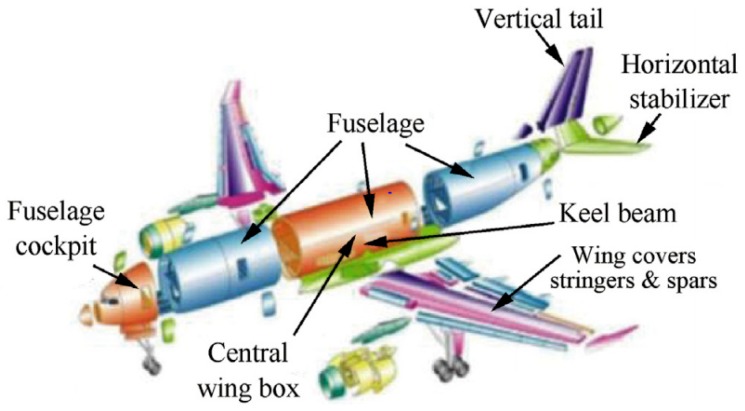
CFRP composite applications in various components of an Airbus 350 (source: Hexcel [93]).

Nano-composite materials have been proved as suitable approaches to enhance airplane strength and safety during operation [92]. Glass fiber reinforced polymer (GFRP), and carbon fiber reinforced polymer (CFRP) are excellent composite materials due to their higher strength and lower weight properties [88,92]. These composite materials have been intensively used over conventional materials in the aerospace industry. CFRP composite shows superior properties with lower weight and higher stiffness [92]. These composite material applications may include airplane components such as doors, windows, wing flaps, etc. as shown in Figure 9 [93].

The airframe is the largest part of the aircraft which protects it from the external environment. Therefore, it should be strong enough to accommodate external friction while it should be light in weight to reduce the overall fuel consumption. For this purpose, nanocomposites are suitable options to improve the strength and mechanical properties of the aircraft [91]. In ref. [94] simulation studies on the performance of the CNTs in the “heavy” commercial aircraft category (heavy aircraft category as defined by the Federal Aviation Administration (FAA)) were conducted. In the study of O’Donnell (see ref. [95]), a carbon nanotube reinforced polymer (CNRP) was used as the airframe material in four simulated aircraft structures (namely the Boeing 757-200, Boeing 747-400, Embraer E145 and Airbus A320). The simulation was performed by using CNRP (70%) to reduce by the same portion the use of conventional aluminum. As a consequence, the results indicated a 14.1% weight reduction and 9.8% fuel reduction in a take-off scenario of the aircraft. The study further indicated that CNRP material could be an optimal option for future aircraft [96]. Veedu et al. [97] investigated a 3D composite with nanotubes to enhance the mechanical properties, thermal and electrical conductivity of aircraft interiors, proving this approach is useful. The use of nanocomposites offers various advantages to ensure the safety and mechanical properties of aircraft.

#### 3.2.2. Nanotechnology Application for the Protection of the Airplane Body

Aircraft icing and lightning strikes are the two most dominant phenomena which demand great attention to ensure the safe operation of aircraft. Aircraft icing occurs upon the presence of the water droplets below the freezing temperatures in the atmosphere that impinge on the aircraft surface during flight operations [98]. These icing effects on the aircraft surfaces could cause stability and control problems during the take-off. Moreover, the icing on the propulsion system components can help decrease the propulsion efficiency and enhances the drag. Research is still ongoing to find the optimal icephobicity for the aircraft. In [99] the study indicated that composite materials reinforced with CNTs could reduce the icing effects on aircraft. In addition, recently, [100] applied a synthesized meso-/macropore carbon nanotube paper (CNP) with a self-healing composite base on the CNP. From the analysis of the results, it was found that this method enhances the electrical conductivity and deicing effect.

On the other hand, the threat of lightning strikes to aircraft is a big challenge to ensure aircraft safety in the atmosphere. This is because a lighting strike on an untreated surface can increase the electrical current value up to 200,000 A [101]. This higher current value can increase the heating of aircraft and ignite the vapors in the fuel storage tanks which threatens aircraft operation. For this purpose, the use of CNTs and carbon nanofillers (CNFs) for the aircraft lighting strike prevention applications are effective techniques to add non-conducting polymers to conductive materials [102]. Moreover, buckypaper which is a macroscopic assembly of entangled CNTs [103] could be applied to ensure higher current carrying capacity during aircraft lightning strikes and is also useful for the protection of the electrical circuits of aircraft [103]. 

#### 3.2.3. Nanomaterials for More Efficient and Effective Body and Wire Networks in Airplanes

**Figure 10:**
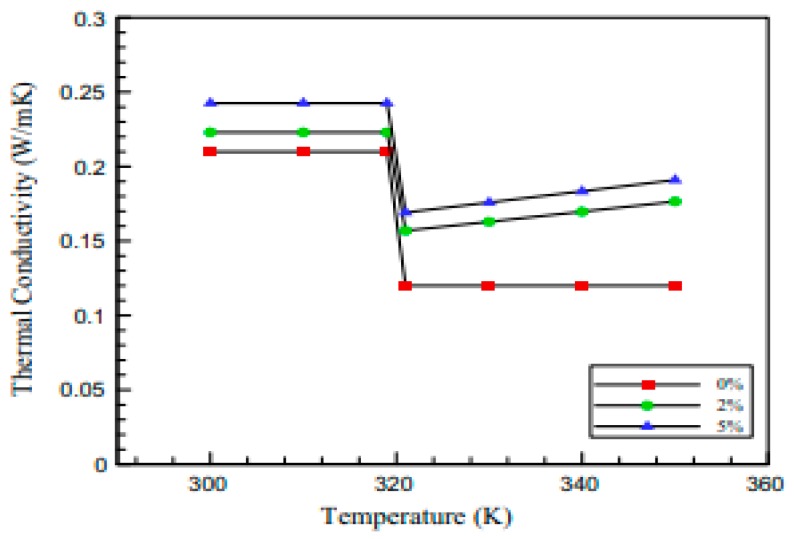
Comparison of the thermal conductivity of a PCM incorporated with nano-architecture (0%, 2%, 5% of Al_2_O_3_) [107].

Nanomaterials can improve thermal performance by decreasing the time constants and diffusion lengths to enhance the power density [104]. For thermal conductivity purposes, phase change materials (PCMs) are beneficial because the thermal loads can be absorbed through the latent heat of the phase change mechanism [105]. However, in order to strengthen the thermal conductivity of the PCM, the incorporation of nanocomposites (Al_2_O_3_) is a very useful approach as shown in Figure 10. Shamberger and Fisher [104] indicated that PCM composite nano-architectures have a higher thermal conductivity than PCMs alone. The greater conductivity of 2–5% ofAl_2_O_3_ helps achieve a larger thermal accessible volume, which minimizes the overheating of the devices [106]. The addition of the nanocomposite in PCMs is a handy technique to equally distribute the heat and enhance the aircraft safe operation during lightning strikes. 

Furthermore, nanocomposite materials enhance the electrical conductivity as well as safety in severe weather conditions [102]. For example, a study [102] indicated that the CNTs and CNFs are very useful to improve the electrical conductivity of the circuits of aircraft. The use of nanomaterials is an effective approach in various parts of the aircraft to improve the thermal as well as electrical conductivity.

#### 3.2.4. Nanotechnology Coatings to Enhance the Sensing and Safety of the Aircraft

**Figure 11:**
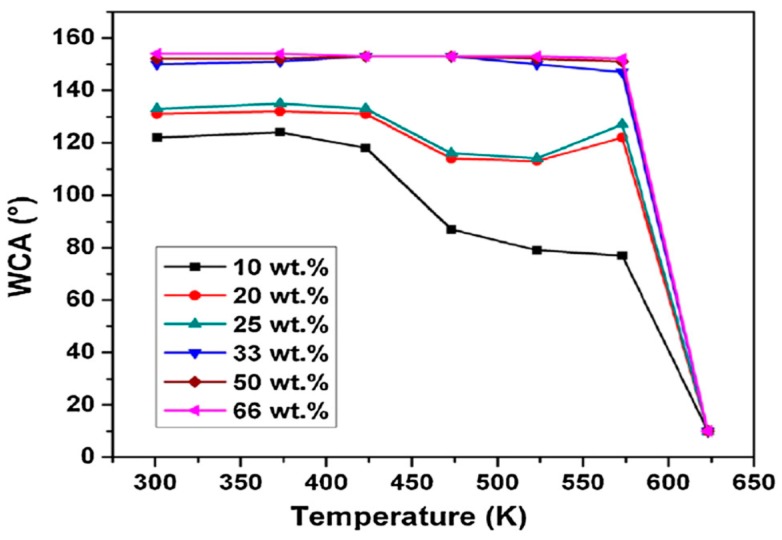
Variation of the water contact angle with temperature in the coatings (Polyvinylidene fluoride and multiwall carbon nanotube [109].

Nanomaterials can display self-healing properties which make them more effective in terms of longer-term sustainability [46,108]. However, in the future, the nanomaterials could identify component damage on time and help prevent failures. Nanotechnology as a sensing technology will be very helpful for the development of future safer aircraft. Additionally, the application of nanotechnology helps to safer operation of the aircraft. This is because nano-based coatings and tires on an aircraft help it resist higher friction and roughness. It can be seen in Figure 11 how nanocoatings help the safer operation of aircraft. 

Figure 11 indicates that the sliding angle (SA) and the water contact angle (WCA) were found to be <3° and 154°, respectively compared to the WCA of polyvinylidene fluoride (PVDF) coating which is 105° (hydrophobic). It is also indicated that the roughness of the coating after addition of multiwall carbon nanotubes (MWCNTs) causes superhydrophobicity [109]. Figure 11 shows the value of WCA measured at different temperatures. It showed that for 20–30 wt% coating, the WCA continuously decreases reaching 10° at 623 K [109]. It is also indicated that the 33 wt% of MWCNT in the PVDF is compulsory for maintaining the superhydrophobicity of coatings. These characteristics of the MWCNT/PVDF make coatings more suited for aerospace use in wet climatic conditions.

Carbon nanotubes are applied for shielding the sensitive parts of airplanes from electromagnetic radiation. In the airplanes, vibrations sometimes cause turbulence and severe vibrations can affect the overall airplane performance. However, the application of nanomaterials can provide high vibration damping properties which can further dissipate the vibration effects through halting slip motion [110,111,112]. On the other hand, airplane surface degradation is another issue of concern in the long term. Various walls of CNTs, SiO_2_, TiO_2_ nanoparticles and graphene in polymeric coatings could be applied as they can lessen cracks over a longer period [91,113]. Despite all the studies mentioned above, there is still a need for low-cost multifunctional nanomaterials for application in the aerospace industry.

### 3.3. Nanotechnology Applications in Marine Transportation

The main purpose of marine transportation is the safe movement of people and other things (cargo, weapons, food, etc.) from one place to another. However, the corrosion of ships is a serious problem in the sea environment. This is because seawater has a high level of salinity which contributes significantly to the corrosion of ships. Stainless steel is an effective material to prevent corrosion in a normal atmosphere; however, in sea water, the atmosphere is entirely different. On the other hand, the erosion and fouling of the bottom of the ship in seawater also affects the performance of ships after a period of time. Adoption of nanotechnology in the ship building is proved as a sustainable approach to improve ship performance over a longer period [114,115]. The potential applications of nanotechnology in ships industry are in corrosion resistant coatings, biofouling, and structural health monitoring.

#### 3.3.1. Nano-Based Coatings to Handle Bio-Fouling and Corrosion

Bio-fouling and corrosion can cause a various adverse effects on ship turbines. As the ships are constantly moving in the seawater which has a high salt proportion it is very difficult to handle bio-fouling on the turbine. Recently, a coating is suggested as an effective strategy to prevent corrosion of the ship in seawater. Keshi et al. [116] reported that CNTs could enhance the wear resistance of the coating. 

**Figure 12:**
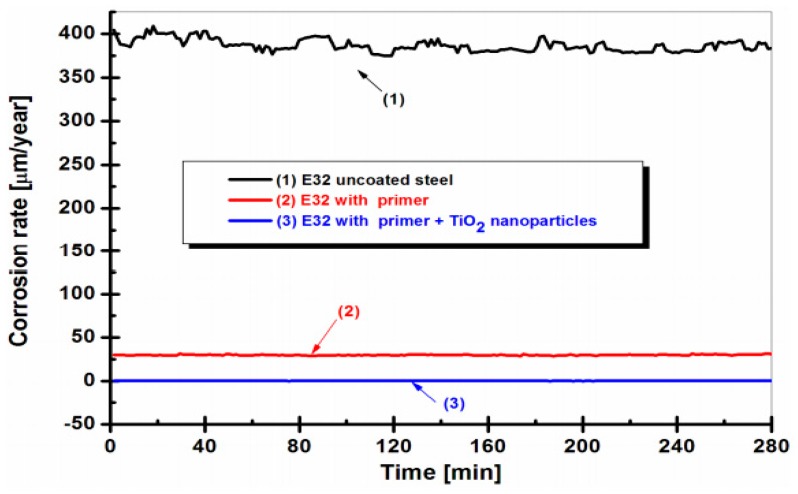
The variation of corrosion rate vs. time of three surfaces in a marine environment [117].

Mardare and Benea [117] evaluated the performance of anticorrosive polymer nanocomposites coatings in the marine environment. For this purpose, they selected naval steel for the experiments in sea water. Three steel surfaces named as E32 uncoated steel; E32 with primer (painting) and E32 with primer + TiO_2_ nanoparticles were prepared to evaluate their performance against corrosion in seawater. From the results, it was found that the corrosion rate was very high in the case of E32 uncoated steel [117]. Contrarily, E32 with primer + TiO_2_ nanoparticles surface layer showed a tremendously lower corrosion rate which indicated that nanoparticle coatings have higher corrosion resistance in seawater as shown in Figure 12.

**Figure 13:**
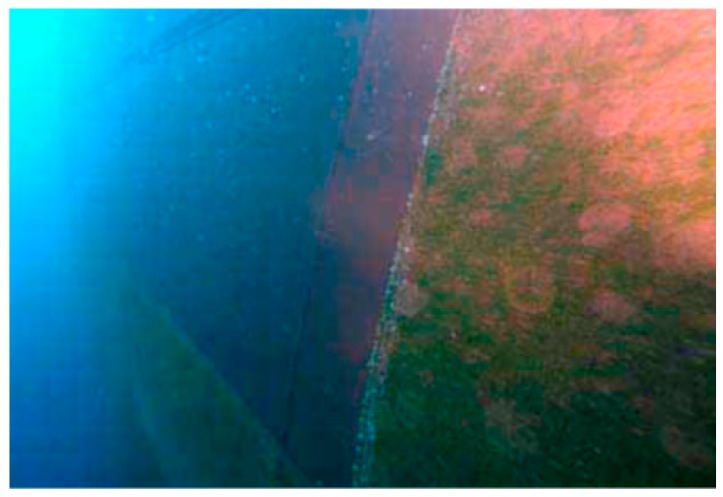
Ship’s hull showing a patch covered with silicon hydrogel (right side), whereas slime and algae are found on the hull with SPC coating paint (left side). Reprinted from [120] with the permission of the American Chemical Society, copyright 2019.

On the other hand, the study described in [118] revealed that the application of carbon nanomaterials as a cathodic coating could reduce microbial fouling. To prevent corrosion in ships, various nanoparticles of TiO_2,_ MgO, ZnO, and Al_2_O_3_ can be mixed into the paint coatings [119]. Ciriminna et al. [120] indicated that the silicon-based coatings provided an effective coating to reduce the fouling in seawater. In the Figure 13 on the left side (with silicon-based coating) the hull of the 380 m long TI Asia Ultra oil tanker free from fouling over a longer interval of time (>13 months) in seawater. However, on the right side, the algae and slime fouling are found because a self-polishing copolymer paint (SPC) coating was applied [120]. To reduce the fouling and corrosion, there is a high need to select the appropriate nanomaterials for coatings purposes.

#### 3.3.2. Nano-Based Materials for the Enhancement of Strength of Marine Vehicles

Nanomaterials are also very useful in enhancing the structural performance during long operation. This is because nanomaterials are the “smart” materials which can display damage sensing and self-healing properties [121]. CTNs possess these multifunctional characteristics which can sense the damaged part of the structures which gives useful information to control the operation failure of the system. Power loss is a severe concern in ship operation which can affect the overall performance of the ship. For this reason, various sensing technologies are used in ships to provide useful information regarding the overall operation of ships. Therefore, if a power loss occurs then it could affect the performance of ships. In [22] it was indicated that carbon nanotubes could be applied to cables/wires to enhance the conductivity. Therefore, the application of nanotechnology could make marine transportation safer over a longer period.

The application of nanotechnology in transportation may become more attractive when it is more cost effective. This can bring the current transportation automobiles to a higher level. The integration of nanotechnology in vehicles can improve the performance of the system directly. However, there is a need to select the most cost-effective as well as an environmental friendly nanomaterial for transportation.

## 4. Environmental Health and Safety Concerns

**Figure 14:**
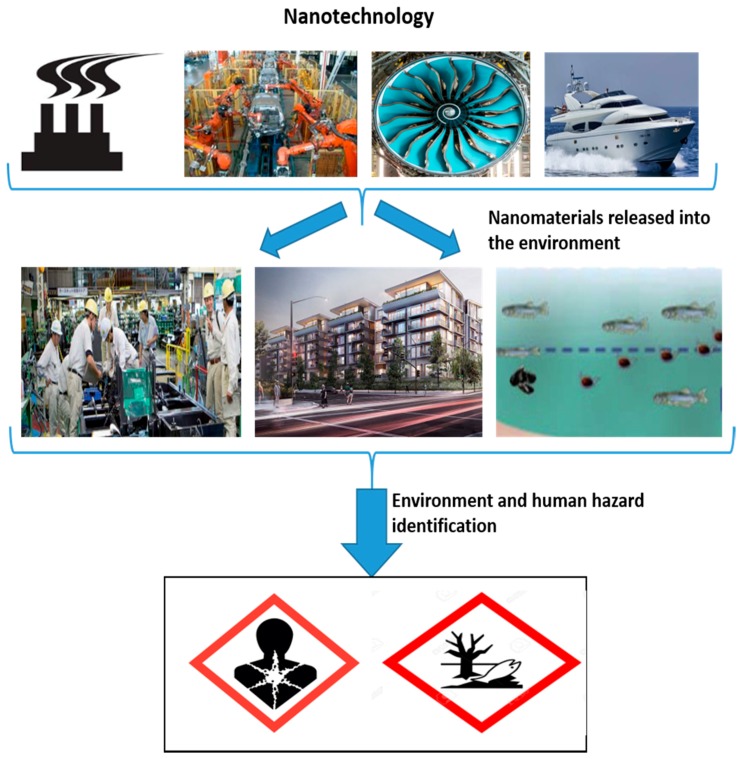
Nanotechnology environmental health and safety concerns.

Recently, the use of nanotechnology and its applications have spread widely due to their numerous advantages at the nanoscale in various science and engineering fields, including transportation vehicles around the world. Nanotechnologies have been applied in the vehicle industry for the enhancement of the efficiency of the vehicles. However, during the manufacturing and use of nanotechnology, there are high chances of nanomaterial exposure for workers and the environment [122]. Such exposure may cause toxic impacts which affect the environment as well as human health [122,123]. Hence, nanoparticles are responsible for the harmful effects on biological organisms, which are also very difficult to identify. The biggest challenge is the assessment of hazards of nanotechnology to humans, animals and the natural environment which could lead to more adverse conditions [124,125]. According to recent estimates [126], around 6 million workers will likely exposed to nanoparticles in 2020. A few of the known nanomaterials with detrimental impacts include TiO_2_ [126], carbon-containing nanomaterials [127,128], Cu and ZnO nanoparticles, etc. [129]. However, the effects of most nanomaterials are still unknown which could cause more adverse effects on the environment as well as living organisms. Moreover, a systematic database of toxic effects and occupational exposure limit (OEL) for nanomaterials is not available all around the world [124,130]. Figure 14 below shows how nanomaterial toxicity and exposure in the various environments could cause harmful effects. Therefore, recently, several concerns have arisen regarding the safety of nanotechnology applications in the real world.

### 4.1. Nanotechnology Environmental and Health Concerns

All the nanomaterials which used in the various parts of vehicles must be adaptable to the environment, and their effects should not be detrimental. Therefore, recently much attention has been paid to finding out the consequences of different nanomaterials on the natural environment. This will help identify the toxic nanomaterials which can be avoided in future nanotechnology applications in the vehicle industry. This will help to create a healthy and environmentally friendly atmosphere in the automotive industry. Shi et al. [131] studied the implications of the nanomaterials used in vehicles. From the research results, it was found that the amount of particles below 10 nm in size that were found at the roadside was more than 40% [131]. The use of nanomaterials has not only increased the concentration of nanomaterials but also broadened the range of nanomaterials in the natural environment [132,133]. Recently, nanomaterials can be found in nano-coated screens and green tires, etc. Various studies have indicated that nanomaterials have an adverse effect on the natural atmosphere as well as on human health [131,134]. For example, carbon nanotubes can pose several hazards to workers such as adverse effects on the lungs, cell membranes and respiratory system [135,136,137]. In addition, other nanomaterials such as TiO_2_, SiO_2_, and Cu have also indicated toxic effects which affect human as well as the other species’ life [138,139]. Hence, the use of nanotechnology in transportation engineering demands particular care. For this purpose, researchers should carefully investigate the use of nanomaterials as well as their consequences to the natural environment over several years. 

#### 4.1.1. Nanoparticle Toxicity

Nanomaterials used in transportation vehicles can cause toxic effects such as inflammation and DNA damage through various stages from manufacturing to recycling [135,136]. Due to their small size, nanomaterials are more active and can easily enter cells and disturb their function. Nanomaterials adversely affect the immune system through immune toxicity, and it can further extend to immunosuppression. The nanomaterials can be easily inhaled, which further affects the respiratory system [137]. TiO_2_ and carbon nanotubes are the most studied nanomaterials because of their potential toxic effects. Table 2 shows the toxic effects of nanomaterials.

CuO nanoparticles are widely used in the coating of boats and ships, etc. However, the toxic nature of CuO adversely affects aquatic life [127,154]. Results indicated that CuO nanoparticles are more toxic as compared to micro-sized CuO and they are around fifteen times more toxic to microalgae [155], and sixty times more toxic to yeast [148]. Reference [156] indicated the enhancement of lipid peroxidation (LP) products (oxidative stress) in mongrel dogs (male) after the addition of 1 mg kg^−1^ C_60_(OH)_18_. This indicated that continuous addition of C_60_ fullerenes causes adverse effects on animal health and could be more dangerous for the human body. Similarly, TiO_2_ and SiO_2_ have been reported as toxic nanomaterials. They can cause various toxic effects such as inflammation, cytotoxicity, and DNA damage. Therefore, much attention is needed for the careful selection of nanomaterials regarding their long term consequences.

#### 4.1.2. Exposure of Nanotechnology

Exposure of living organisms to nanomaterials can be inimical to their health. The two main causes for their harmfulness are: (1) their small size, which allows nanomaterials to penetrate to the living cells and disturb their function; and (2) nanomaterials can enter tissues, including the brain and can affect their function. Nanomaterial exposure can cause several negative effects, such as inflammation of tissues, cytotoxicity, oxidative stress, DNA damage, and neurological and other diseases, etc. Several studies [157,158,159,160] have identified the respiratory system as the main exposure route for nanomaterials [157]. However, nanomaterials can also enter through the skin and eyes. Dermal absorption and inhalation of nanomaterials are the two main concerns which can affect workers’ health seriously over the long term. This is due to the small size of nanomaterial which can easily form aerosols that can easily reach the lungs and affect their function [157]. Another issue of nanomaterials is that they can translocate to the organ system via the lymphatic and blood system and cause direct or indirect harm [157]. Another large part of the body which can be affected by nanomaterials is the skin. Larese et al. [158] indicated the potential risk of the dermal exposer due to nanomaterials in the workplace. Air pollution problems in the working environment through exposure to nanomaterials needs to be addressed by proving a hazard exposure limit. Several studies [32,33,135] have indicated the detrimental effects of nanomaterials on workers’ health on the job site. The detected effects include oxidative stress [140], inflammation problems [136], heart problems [159], skin problems [158] and lung function problems [161]. In the future, there is a need to carry out more research to identify the potential risks of using nanomaterials on human health. 

**Figure 15:**
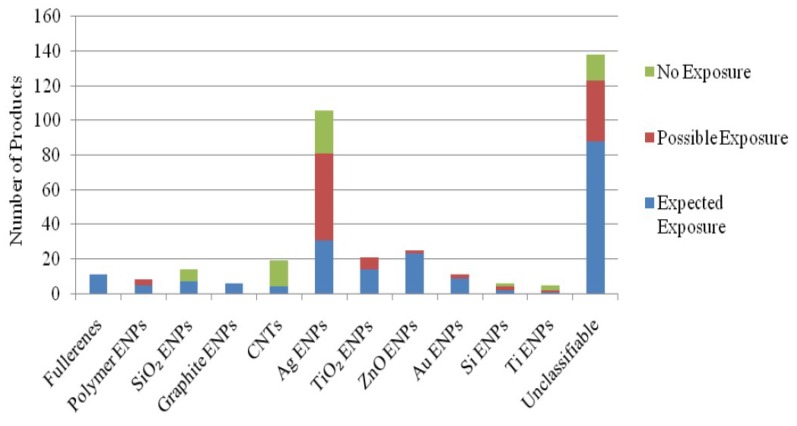
Nanomaterials versus likelihood of exposure [160].

Foss et al. [160] compared the potential risk of exposure of several materials against several nanomaterials as shown in Figure 15. The study indicated that a higher number of products are unclassified products and have higher potential for consumer exposure. Therefore, the lack of information about nanomaterial exposure can be potentially hazardous for consumers. A challenge with the measurement of the hazard of any nanomaterial is that the physico-chemical parameters of nanomaterials are not yet known well enough to control the limits of nanomaterial hazard exposure limits to the human body.

#### 4.1.3. Impacts on Human Health

Experimental studies on nanomaterials suggest that ultra-fine sized particles in the air have a significant effect on the respiratory and cardio-respiratory diseases [162]. A recent study [163] was carried out in Korea where the workers were exposed to MWCNTs. Research indicated local and systemic markers of pulmonary damage in the workers at the site. Another study [164] was also performed in Taiwan to figure out the effect of nanomaterials on exposed workers. From the research results, it was found that there was an increasing number of cardiovascular diseases in the workers exposed to nanomaterials at the site. Therefore, there is a need for more experimental work to find toxic nanomaterials at manufacturing sites. The major concerns about nanomaterials in the transportation industry are indirect exposure due to the migration of nanosized particles from automobile bodies. Thses will degrade the natural environment and cause serious human health problems. On the other hand, nanoencapsulation allows direct contact of nanoparticles through the intake of oxygen to the body. SiO_2_ and CNTs are the most widely used nanomaterials in the automotive industry to enhance strength and durability [14,91]. However, their longer-term toxicity and exposure to human are still uninvestigated in the real world [130]. Continuous releases of nanomaterials in the working environment could cause various diseases to workers. Therefore, in the future, the ultimate fate and toxicity of nanomaterials should be noted for their safer application. Safe application of nanotechnology to the transportation industry requires a thorough assessment of nanomaterials’ characteristics in vitro, and in vivo [33]. Moreover, one also needs to consider other factors such as physical forces, pH, chemical factors, their absorption, metabolism, distribution, excretion, exposure, and toxicity that could be quantified and evaluated for risk assessment [165]. For safer nanotechnology application, there is a high need for proper public education about its applications and environmental concerns. Besides, there are a number of nanomaterials which can have adverse effects on human health [166]. For example, several nanosized metal oxides cause inflammation, toxicity and oxidative stress in the human body. Another study by [167] indicated that the C_60_ fullerenes also accumulate in the liver and affect its function. Nanosized particles easily can enter the human skin and can cause several diseases in the human body [168]. Currently, there is a need to find more specific methods to evaluate the adverse effects of nanomaterials on the environment as well on the human body.

### 4.2. Safety Concerns

As already mentioned the application of nanotechnology in the vehicle industry enhances the materials’ functions as well their long term durability. However, there are significant safety concerns regarding the application of nanotechnology, including human exposure and toxicity [169,170,171]. It is also inevitable that human exposure to nanoparticles will increase in the future in various ways. Till now, there were very few experimental studies [172,173,174] that have focused on the exposure and potential toxicity of nanomaterials to natural environments as well as living organisms. There is little known information related to the routes of exposure, the limits of nanomaterial exposure, and the toxicity of nanomaterials in occupation-related scenarios around the world. For example, nanoforms can exhibit different fate and hazard behavior and thus different risks. Therefore, in the real world, it is challenging to address all the nanoforms which leads to a research gap regarding the safety policy considerations [172]. Moreover, due to the physicochemical properties of nanoparticles, their potential threat may change during their Life Cycle Assessment (LCA) [175]. This issue has been considered by several researchers during their life cycle risk assessments of nanomaterials [176,177,178]. Under certain conditions, the production of hazardous material during the life cycle requires great attention. For example, the coating disintegration could lead to more complexity regarding safety. Other methods such as standardized testing, benchmarking of materials and in silico approaches have been applied for risk assessment [172]. However, there is a still need for developing globally accepted rules/guidelines, especially for the surface affinity and surface reactivity of nanomaterials to address their specific behavior and hazards in the real world [172].

**Figure 16:**
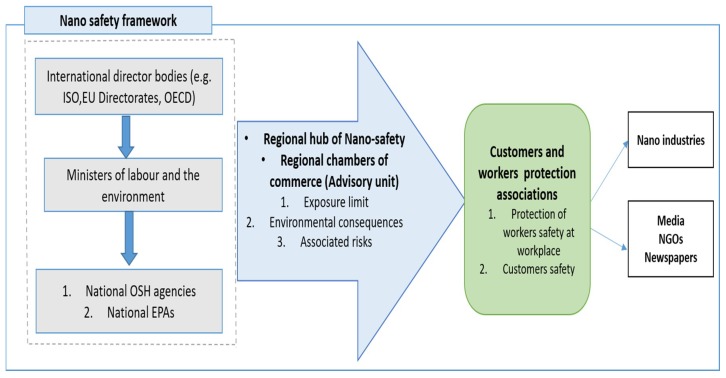
Nano-safety framework for enhanced safety in the nano-industry. Adopted from [180].

In the real world, environmentally aware product design frameworks and life cycle assessment strategies need to be set comprehensively for the enhancement of nanosafety to the environment. To specifically characterize and track nanomaterials in the environment, the most critical steps needed to investigate further in details are the formulation of nanomaterials databases to investigate the LCA of nanoproducts; gathering the real data of nanomaterials for the development of exposure limits of nanomaterials as well as formulation of advanced models; development of standardized protocols for handling nanomaterials in the workplace and the development of risk assessment methods to enhance their overall safety [179,180]. These steps will help assess the risks related to the application of nanomaterials in the real world, which hence can further enhance the safety in the workplace. At the national level and international level, there is a great demand to set up a systematic strategy framework which can prevent risks and alleviate the safety concerns associated with each nanomaterial for both workers and customers [29,181,182]. Figure 16 below presents the national strategic framework which can provide useful information of each nanomaterial to the nano-industry to enhance the safety of workers and customers [180]. By considering the above EHS challenges, a suitable expertise group within government ministries could provide an appropriate framework for the safer and sustainable application of nanotechnology as shown in Figure 16. This strategy utilizes useful information of experts to develop a national database system which can provide an EHS risk awareness for nanomaterial-developing nations [180,183]. This proactive technique can provide the expert information in a timely way to manufacturers and consumers effectively and efficiently.

## 5. Outlook for Future Research

**Figure 17:**
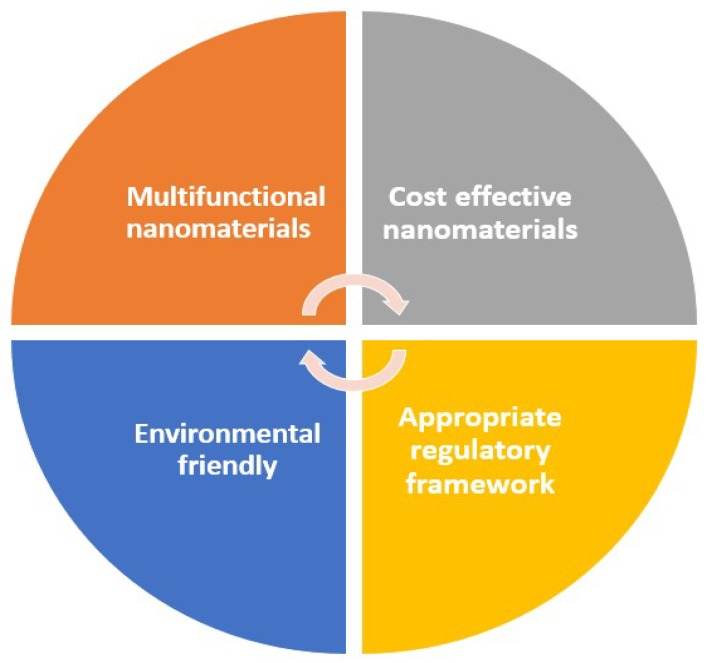
Major four research gaps and future opportunities which need to be considered for the broad and sustainable application of nanotechnology in transportation vehicles.

Even though nanotechnology in the transportation vehicle industry manifests extremely attractive characteristics for safer and sustainable use, however there are still research gaps and opportunities for future work. This section of the study mainly focuses on the most important research gaps and future directions in the field of nanotechnology in the vehicle industry. Four main research gaps that still exist were observed that demand high attention for future safer application as shown in Figure 17.

### 5.1. Cost Effective Nanomaterials

As discussed in Section 3.1.2 and Section 3.2.1, great progress has been made in the enhancement of nano-based materials’ performance for transportation vehicles. The adoption of nanotechnology in the vehicle industry requires a large cost to obtain the required functions. However, one of the important gaps identified is a lack of studies to find the most cost-effective materials for the vehicle industry (i.e., automobiles, marine vessels, aerospace). Furthermore, as reported in [19] the overall costs of the vehicles could be decreased by selecting the most appropriate materials during the manufacturing process. In the real fields, there is a lack of studies about cost-effective nanomaterials in the vehicle industry. Therefore, there is a high need to identify cost-effective nanomaterials for this industry. This could be done through the life cycle assessment of the various nanomaterials which will help to select the most cost-effective nonmaterias for future development.

### 5.2. Multifunctional Nanomaterials

As reported in some previous studies [38,184], CNT-based multifunctional coatings could enhance the safety and durability of vehicles over a longer period of time. However, we lack studies that address the application of multifunctional nanomaterials for multiple benefits in the transportation vehicle industry. Therefore, there is an opportunity for experimental studies to evaluate the benefits of nanomaterials in vehicles, selecting the most appropriate nanomaterials for future efficient and effective vehicles. In the future, there will be a high demand for the selection of multifunctional nanomaterials with several benefits in transportation vehicles around the world.

### 5.3. Appropriate Regulatory Framework

As discussed in Section 4.1.1, Section 4.1.2 and Section 4.1.3, nanotechnology has serious EHS concerns in the vehicle industry. Several studies [135,146,150,161,185,186] have indicated that nanotechnology applications have adverse effects on human health as well as on the natural environment. Most of the nanomaterials are emitted to the natural environment and are usually responsible for serious worker health issues [166,167,187]. Till now, there are no specific exposure limits for nanomaterials in the various nano-based industries which is an alarming situation in this developed world. There is also a need for further studies to find out the safe exposure limits of nanomaterials. These steps will not only help to reduce the harmful impact of nanomaterials, but also encourage the safe application of nanomaterials around the world [181,188]. Moreover, there is no specific framework which can regulate the potential hazardousness of nanomaterials. In addition, the exposure of humans as well as the environment to nanomaterials is increasing, and hence a possible cautionary step needs to be taken to reduce the harmful effect of nanomaterials [182,189]. Therefore, there is a need for a huge amount of work to define an appropriate regulatory framework for nano-based industries. This could be done through wide experimental work to know the nature of nanomaterials. Then there should be an appropriate regulatory framework which sets exposure limits, and defines the toxicity of nanomaterials. This framework should include detailed information about the nanomaterials used in each industry from manufacturing to end of life.

### 5.4. Environmentally Friendly Nanotechnology

As discussed in Section 4.1, there is a huge need for environmentally friendly nanomaterials for a future safer and clean environment. Numerous studies [13,166,167,187] have indicated the toxicity of the nanomaterials which could affect living organisms as well as the natural environment [190]. To identify environmentally friendly nanomaterials, there is a need for extensive field experimental data such that a comprehensive and robust behavioral and nature of nanomaterials database can be created, through which we can know about the nature and performance of nanomaterials over a period of time. As a result of this, we can select the most environmentally friendly nanomaterials for future efficient transportation vehicles. These steps need to be followed with great attention for the successful application of nanotechnology in the transportation field.

## 6. Conclusions

The application of nanotechnology in vehicles presents a great range of opportunities for researchers. By introducing nanotechnology in the transportation vehicles industry, we can make vehicles smarter, more efficient, stronger and durable. This paper reviews the literature regarding the evolutionary changes in transportation vehicles achieved by introducing nanotechnology, and the associated environmental health and safety concerns for future large scale application of nanotechnology in the vehicle industry. In the automotive industry, the overall performance of paint coatings, engines, body parts, mirror, tires, etc. are enhanced by the incorporation of various nanomaterials like CNTs, TiO_2_, SiO_2_, and carbon black. In addition, higher strength, lightweight, flame and fire and UV resistance of aerospace materials are tremendously enhanced by the application of nanomaterials. Moreover, the corrosion and fouling can be reduced in ships by applying nanomaterial coatings. Although many great features such as comfort, safety, and durability related to nanotechnology applications have been reported, there are many factors which are still unknown.

This paper also reviews the environmental health and safety concerns regarding nanotechnology applications in the vehicle industry. Nanomaterials’ toxicity and exposure are the two main concerns that demand great attention to create a clean and healthy environment. As nanomaterials are new products in the transportation vehicle industry, it is essential to understand their potential impacts in and across air, soil, and water. More in-depth research work on environmental friendly nanotechnology is required, and multidisciplinary research cooperation and collaboration in dealing the environmental challenges are imminent. Future studies could focus more on the adoption of environmental friendly nanotechnology in various industries. For this purpose, it is necessary to incorporate the multiple aspects of nanotechnology applications in the real world. An honest analysis of the nanotechnology application methodologies, services, and environmental impact would be beneficial in helping communities identify what nanotechnologies are environmentally sound and safe for future development. Besides, there is no regulatory framework regarding the exposure to nanotechnology at the workplace. There is also very limited knowledge about the long term effect of nanotechnology products in various fields in real world applications. Understanding how nanomaterials affect multiple scenarios, as well as exposure in the future is essential. By developing an effective impact assessment system, we could be assured of the safer and sustainable application of nanotechnology in the vehicle industry. We believe that this review study will encourage future research on the discussed topic.

## Figures and Tables

**Table 1:** Experimental studies of nanofluids for vehicle system cooling.

Reference	Nano Fluids Types	Advantages
[65]	Al_2_O_3_−EG	Al_2_O_3_−EG enhances the thermal conductivity by about 4.5% with addition of Al_2_O_3_ nanoparticles (1.5 vol%).
[67]	Nanodiamond–engine oil	Nanofluid enhances the engine performance by increasing the engine power by about 1.15% and reducing the fuel consumption by about 1.27% compared to simple engine oil.
[73]	Al_2_O_3_−water, Al_2_O_3_−EG, Al_2_O_3_−EG/water (5–20 vol% of EG)	Heat transfer performance was enhanced about 40% with the addition of 1.0 vol% of nanoparticles of Al_2_O compared to the pure fluid.
[72]	Al_2_O_3_−water	The maximum improvements of coolant heat transfer coefficient, heat transfer rate and Nusselt number were 14.7%, 14.8%, and 9.5%, respectively.
[69]	CuO−water, Fe_2_O_3_−water	0.65 vol% CuO−water nanoparticles enhanced the heat transfer coefficient by up to 9%.
[70]	CuO−water	CuO−water is beneficial to improve the overall heat transfer coefficient. With 0.4 vol% CuO concentration of nanofluid the heat transfer coefficient was enhanced about 8% as compared to pure water.
[71]	SiO_2_−water, TiO_2_−water	The maximum Nusselt number improvements for SiO_2_ and TiO_2_ nanofluids were 22.5% and 11%, respectively
[74]	SiO_2_−water	With 0.4 vol% of SiO_2_ nanoparticles at 60 °C the heat transfer enhancement was about 9.3% as compared to the pure fluid.

**Table 2:** The toxicity of the various nanomaterials.

Nanomaterials	Toxic Effects	References
Carbon nanotubes	Antibacterial	[139,140,141]
	Damage of cell membrane	
	necrosis/apoptosis	
	Hinder the respiratory functions	
	DNA damage	
	Induce granulomas and atherosclerotic lesion	
	Lung damage	
SiO_2_	Slightly toxic effect	[139,142,143]
	Toxic to marine algae	
	Apoptosis	
	Up-regulation of tumor necrosis factor—alpha genes	
	Inflammatory and immune responses	
C_60_ derivatives	Bactericidal for Gram-positive bacteria	[140,144]
	Oxidative cytotoxicity	
	Accumulation in liver	
	Induces gliomas, sarcomas in mice as well as in human cells	
Quantum dots	Bacterial toxicity	[141,145,146]
	Partials uptake and damage to DNA	
TiO_2_	Growth inhibition and acute lethality	[123,147,148]
	Bactericidal for gram-positive bacteria	
	Elimination of photosynthetic activity	
	Oxidative damage due to ROS	
	Liver damage	
CuO nanoparticles	Freshwater algae toxicity	[149,150,151,152,153]
	Yeast toxicity	
	Damaging DNA	
	Acute toxicity to kidney, spleen, and liver	
